# Immune Regulation in the Male Genital Tract

**DOI:** 10.1155/S1064744996000294

**Published:** 1996

**Authors:** Steven S. Witkin, Jan Jeremias, Ann Marie Bongiovanni, M. Gladys Munoz

**Affiliations:** 1 Division of Immunology and Infectious Diseases Department of Obstetrics and Gynecology Cornell University Medical College 515 East 71st Street New York New York 10021 USA; 2 Departamento de Biologia de Organismos Universidad Simon Bolivar Caracas Venezuela

## Abstract

Spermatozoa are not produced until puberty, long after the establishment of tolerance to self-antigens. Therefore, sperm-specific antigens are immunogenic in men. Most men, however, do not produce antibodies to their own gametes. Development of mechanisms to prevent or limit autoimmune responses to spermatozoa were essential for preservation of reproductive capacity. Tight junctions between adjacent Sertoli cells, as part of the blood-testis barrier, prevent sperm-immune cell contact. In some portions of the genital tract this barrier is thin or incomplete. Immune mechanisms have evolved to actively suppress the autoimmune response to spermatozoa within the genital tract. Unlike in the circulation where CD^4+^ helper T lymphocytes predominate, CD^8+^ suppressor/cytotoxic T lymphocytes are the most prominant T cells in the epididymis and vas deferens. In addition, spermatozoa suppress pro-inflammatory lymphocyte immune responses, possibly by inducing production of anti-inflammatory cytokines. Antisperm antibody production is induced in the male genital tract when a local infection or disruption in the genital tract physical barrier leads to an influx of CD^4+^ T cells. In response to induction of a productive immune response, two additional mechanisms downregulate humoral immunity within the genital tract. T lymphocytes possessing the γσ form of the antigen receptor (γσ T cells) are concentrated in the male genital tract and in semen. These cells become activated and proliferate in men with evidence of sperm autoimmunity. Activated γσ T cells inhibit production of antibodies by activated B lymphocytes, thereby limiting antisperm antibody production. Heat shock proteins (hsps) are also present in semen in association with infection and antisperm antibody formation. Hsp gene transcription leads to inhibition of transcription of the genes coding for pro-inflammatory cytokines and, conversely, to activation of γσ T cells. Activated γσ T cells also promote hsp synthesis. The mechanisms to inhibit immunity to sperm may hinder effective immune elimination of microoganisms in the male genital tract.


Infectious Diseases in Obstetrics and Gynecology 4:131–135 (1996)
© 1996 Wiley-Liss, Inc.

### Immune Regulation in the Male Genital Tract

Steven S. Witkin,[1] Jan Jeremias,[1] Ann Marie Bongiovanni,[1] and M. Gladys Munoz[2]

[1]*Division of Immunology and Infectious Diseases, Department of Obstetrics and Gynecology, Cornell University Medical College, New York, New York;* [2]*Departamento de Biologia de Organismos, Universidad Simon Bolivar, Caracas, Venezuela*

### Abstract

**Spermatozoa are not produced until puberty, long after the establishment of tolerance to self-antigens. Therefore, sperm-specific antigens are immunogenic in men. Most men, however, do not produce antibodies to their own gametes. Development of mechanisms to prevent or limit autoimmune responses to spermatozoa were essential for preservation of reproductive capacity. Tight junctions between adjacent Sertoli cells, as part of the blood-testis barrier, prevent sperm-immune cell contact. In some portions of the genital tract this barrier is thin or incomplete. Immune mechanisms have evolved to actively suppress the autoimmune response to spermatozoa within the genital tract. Unlike in the circulation where $CD4^+$ helper T lymphocytes predominate, $CD8^+$ suppressor/cytotoxic T lymphocytes are the most prominant T cells in the epididymis and vas deferens. In addition, spermatozoa suppress pro-inflammatory lymphocyte immune responses, possibly by inducing production of anti-inflammatory cytokines. Antisperm antibody production is induced in the male genital tract when a local infection or disruption in the genital tract physical barrier leads to an influx of $CD4^+$ T cells. In response to induction of a productive immune response, two additional mechanisms downregulate humoral immunity within the genital tract. T lymphocytes possessing the γδ form of the antigen receptor (γδ T cells) are concentrated in the male genital tract and in semen. These cells become activated and proliferate in men with evidence of sperm autoimmunity. Activated γδ T cells inhibit production of antibodies by activated B lymphocytes, thereby limiting antisperm antibody production. Heat shock proteins (hsps) are also present in semen in association with infection and antisperm antibody formation. Hsp gene transcription leads to inhibition of transcription of the genes coding for pro-inflammatory cytokines and, conversely, to activation of γδ T cells. Activated γδ T cells also promote hsp synthesis. The mechanisms to inhibit immunity to sperm may hinder effective immune elimination of microoganisms in the male genital tract.** © 1996 Wiley-Liss, Inc.

Key words

γδ T lymphocytes, heat shock protein, antisperm antibodies, spermatozoa

The immune system within the male genital tract has been subjected to unique evolutionary pressures. Spermatozoa are not produced until puberty, long after establishment of tolerance to self-antigens during the neonatal period. Therefore, sperm-specific antigens are immunogenic in men. The fact that the majority of men do not produce antisperm autoantibodies indicates the existence of

Address correspondence to Dr. Steven S. Witkin, Department of Obstetrics and Gynecology, Cornell University Medical College, 515 East 71st Street, New York, New York 10021.

Supported in part by NIH grant HD 33194

Received September 15, 1996
Accepted October 1, 1996

Article mechanisms to limit immune responsiveness in the male genital tract. This communication will detail the various means both passive and active by which productive immunity is impaired at this site. This subject has been reviewed previously[1,2].

### BLOOD-TESTIS BARRIER

The first line of defense against sperm-specific autoimmunity is anatomical, the blood-testis barrier. A tight network of intercellular junctions between Sertoli cells retards the passage of sperm-related macromolecules and cells out of, and immune components into, the testis[3]. This physical barrier is narrow in some regions and is not completely effective in preventing an interaction between intra and extra - genital tract components. In mice, T lymphocytes that had been sensitized to spermatozoa and subsequently transferred to recipient males, gained access to the genital tract and reacted with sperm autoantigens[4]. In addition, the integrity of the genital tract can be compromised as a consequence of testicular trauma, inflammation of infectious or noninfectious origin, congenital anomoly or due to the deliberate severing of the vas deferens (vasectomy).

### SUPPRESSION OF IMMUNE RESPONSES

The T lymphocytes that recognized sperm autoantigens in the study cited above were $CD4^+$ cells. These lymphocytes do not react with native antigens. Only those antigens that have been digested and subsequently transported and presented on the surface of an antigen-presenting cell (APC) in association with class 2 major histocompatibility (MHC) molecules can be recognized by the $CD4^+$ cell. Macrophages, one of the major APC, are present in the male genital tract[4]. In their resting state, macrophages do not normally express MHC class 2 antigens. However, interferon gamma (IFN$\gamma$), a product of activated T cells, is a potent inducer of MHC class 2 antigen expression on macrophages[5]. Therefore, one means of inhibiting immune responses within the genital tract would be to prevent IFN$\gamma$ production.

Evidence exists for several mechanisms that inhibit pro-inflammatory, IFN$\gamma$-producing immune responses in the male genital tract. Detailed immunohistological studies of the human male genital tract, summarized by El-Demiry and James[6], demonstrated the predominance of T lymphocytes with the suppressor/cytotoxic phenotype (CD8) within the epithelium and lamina propria of the vas deferens, epididymis, and rete testis. Macrophages were also identified at these same sites as well as between the seminiferous tubules. The authors noted the accumulation of $CD8^+$ T cells in those regions of the tract where the blood-testis barrier were weakest and hypothesized that $CD8^+$ cells may function to actively suppress immune responses to sperm-specific antigens. In marked contrast, $CD4^+$ T cells predominated in both the genital tract[6] and in semen[1] in men with evidence of autoimmunity to sperm. Mononuclear cells present in human semen were also shown to function as suppressor cells in vitro[8]. Further evidence for an active suppression of sperm-directed immune responses comes from studies in mice. Neonatal thymectomy resulted in the subsequent development of orchitis in a process prevented by the infusion of spleen-derived lymphocytes from untreated controls[9].

### SPERM-INDUCED IMMUNE SUPPRESSION

Purified human spermatozoa, when incubated in vitro with peripheral blood mononuclear cells, induce neither lymphocyte proliferation nor the synthesis of IFN$\gamma$. In contrast, sperm with bound autoantibodies are potent IFN$\gamma$ inducers[10]. Similarly, circulating IFN$\gamma$ was identified in sera of women whose husbands had sperm autoimmunity but not in sera from women whose serum were antibody-free[11].

Spermatozoa appear to actively suppress immune responses. Intravenous injection of spermatozoa or testicular germ cells into syngeneic mice profoundly suppressed subsequent immunity by a mechanism involving activation of suppressor T cells[12,13]. Rectal insemination of semen into rabbits led to similar consequences[14]. Similarly, purified, living ejaculated human spermatozoa inhibited in vitro lymphocyte proliferative responses[15]. A failure in sperm-induced immune suppression by their male partners' semen has also been associated with antisperm antibody formation in the female sexual partners.[15]. Recent evidence from our laboratory indicates that human spermatozoa induce the production of anti-inflammatory cytokines by lymphoid cells (in preparation).

### $\gamma\delta$ T LYMPHOCYTES

Recently, T lymphocytes possessing a T cell receptor heterodimer complex composed of $\gamma$ and $\delta$

chains (γδ T cells) have been found to be concentrated in human semen[16,17]. Unlike in the circulation where γδ T cells were less than 10% of the total T lymphocyte population, in semen they comprised 33–50% of the T cells. A greatly elevated seminal γδ T cell concentration was consistently observed in men with sperm autoimmunity or immunological evidence of a genital tract *Chlamydia trachomatis* infection. γδ T cells are also present in the genital tracts of male mice where they have been shown to prevent pro-inflammatory immune resonses to sperm-specific antigens[18]. Similarly, γδ T cells limit both destructive immune responses during experimental infection[19] and maternal anti-fetal immunity during pregnancy[20].

The mechanism of γδ T cell immune suppression may involve the synthesis by these cells of anti-inflammatory cytokines[20,21]. Alternatively, resting γδ T cells can be stimulated by activated antibody-producing B lymphocytes, but not by resting B cells. Once activated, γδ T cells actively suppress the release of IgG from the B cell surface[22]. Thus, the concentration of γδ T cells in the male genital tract provides another mechanism to downregulate immune responses at that site.

### HEAT SHOCK PROTEINS

In response to immune activation and the release of pro-inflammatory cytokines the genes coding for heat shock proteins are transcribed[23]. Extracellular phospholipase $A_2$ activity, an early consequence of microbial infection, also induces heat shock protein biosynthesis[24]. Heat shock proteins prevent intracellular protein degradation and incorrect polypeptide assembly when the cell encounters conditions unfavorable to its survival. In addition, initiation of heat shock protein gene transcription inhibits transcription of the macrophage genes coding for interleukin-1 (IL-1) and tumor necrosis factor-α (TNFα)[23,25,26]. Thus, up-regulation of heat shock protein synthesis is another means of down-regulating pro-inflammatory immune responses.

The 60kD heat shock protein has been identified in semen samples from some men with immunological evidence of a chlamydial infection or sperm autoimmunity but not in semen from men lacking evidence of local immune activation[27]. This suggests that heat shock protein gene responses may be operational within the male genital tract. The ability of γδ T cells to initiate heat shock protein gene expression[28] and, in turn to be activated by heat shock protein[29], coupled with the observed association between seminal γδ T cell concentration and genital tract immune activation[(16,17], reinforces the plausibility of this mechanism.

### REGULATION OF IMMUNE RESPONSES IN THE MALE GENITAL TRACT.

Based on the experimental evidence cited above, we propose the following mechanism of immune regulation in the male genital tract (Fig. 1). In the case of men with neither sperm autoimmunity nor a genital tract infection, contact between spermatozoa and T cells and macrophages does not lead to a pro-inflammatory immune response. IFNγ is not being produced due to the lack of immune stimulation. MHC class 2 expression on macrophages is, therefore, not induced. Concurrently, spermatozoa induce the production of anti-inflammatory cytokines. Therefore, genital tract immunity is down-regulated. Conversely, however, in men with pre-existing sperm autoimmunity or a current genital tract infection, activation of $CD4^+$ αβ T cells induces the release of IFNγ. This activates macrophages to engulf spermatozoa, transport spermatozoal antigens to the macrophage surface in association with newly expressed MHC class 2 molecules. The activated macrophage also releases the pro-inflammatory cytokines IL-1 and TNF-α This overwhelms the pre-existing immune suppression and B lymphocytes are activated to produce antibodies to spermatozoal and microbial antigens.

Should a pro-inflammatory immune response be initiated its magnitude is down-regulated at discreet stages by the mechanisms also shown in Fig. 1. Pro-inflammatory cytokine synthesis initiates heat shock protein gene transcription. Concomitantly, antibody-producing activated B cells stimulate the proliferation of γδ T cells. Heat shock protein also stimulates γδ T cells activation and vice versa. The activated γδ T cells then inhibit further antibody production while heat shock protein gene transcription inhibits further transcription of inflammatory cytokine genes.

An intriguing question still open to speculation is whether the capacity of some men to effectively combat infections at this site is impaired by the evolution of these mechanisms which limit sperm autoimmunity in the genital tract, or by differences

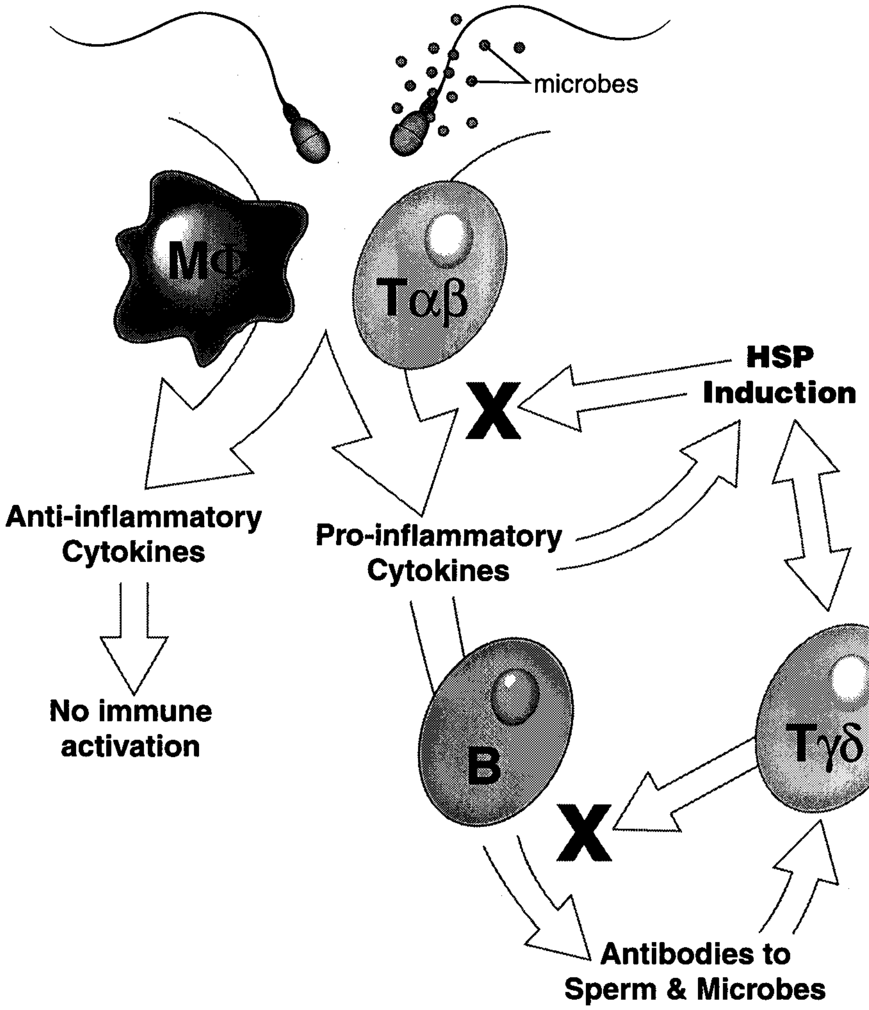

Fig. 1. Regulation of immune responses in the male genital tract. In the absence of infection spermatozoa do not activate pro-inflammatory immune responses from macrophages (Mϕ) or CD4$^+$ αβ T cells (Tαβ). When the male genital tract is infected pro-inflammatory cytokines are induced and B lymphocytes (B) produce antibodies. γδ T cells (Tγδ) are stimulated by activated B cells and inhibit further antibody production. Pro-inflammatory cytokines and γδ T cells initiate heat shock protein (HSP) gene transcription which down-regulates further pro-inflammatory cytokine biosynthesis.

in the magnitude of these responses due to genetic variability, or some combination of both.

### ACKNOWLEDGMENT

We thank Dr. Andreas Neuer for critical discussions

### REFERENCES

1. Witkin SS: Mechanisms of active suppression of the immune response to spermatozoa. Am J Reprod Immunol Microbiol 17:61–64, 1988.
2. Mahi-Brown CA, Yule TD, Tung KSK: Evidence for active immunological regulation in prevention of testicular autoimmune disease independent of the blood-testis barrier. Am J Reprod Immunol Microbiol 16:165–170, 1988.
3. Dym M, Fawcett DW: The blood-testis barrier in the rat and the physiological compartmentation of the seminiferous epithelium. Biol Reprod 3:308–326, 1970.
4. Tung KSK, Yule TD, Mahi-Brown CA, Listrom MB: Distribution of histopathology and Ia positive cells in actively induced and passively transferred experimental autoimmune orchitis. J Immunol 138:752–759, 1987.
5. Beller DI: Functional significance of the regulation of macrophage Ia expression. Eur J Immunol 14:138–143, 1984.
6. El-Demiry M, James K: Lymphocyte subsets and macrophages in the male genital tract in health and disease. Eur Urol 14:226–235, 1988.

7. Witkin SS, Goldstein M: Reduced levels of T suppressor/cytotoxic lymphocytes in semen from vasovasostomized men: relationship to sperm autoantibodies. J Reprod Immunol 14:283–290, 1988.
8. Witkin SS: Suppressor T lymphocytes and cross-reactive sperm antigens in human semen. AIDS Res 1:339–345, 1984.
9. Taguchi O, Nishizuka Y: Experimental autoimmune orchitis after neonatal thymectomy in the mouse. Clin Exp Immunol 46:425–434, 1981.
10. Witkin SS: Production of interferon gamma by lymphocytes exposed to antibody-coated spermatozoa: a mechanism for sperm antibody production in females. Fertil Steril 50:498–502, 1988.
11. Witkin SS, Chaudhry A: Circulating interferon-γ in women sensitized to sperm: new mechanisms of infertility. Fertil Steril 52:867–869, 1989.
12. Hurtenbach U, Shearer GM: Germ cell-induced immune suppression in mice. Effect of inoculation of syngeneic spermatozoa on cell- mediated immune responses. J Exp Med 155:1719–1729, 1982.
13. Hurtenbach U, Morgenstern F, Bennett D: Induction of tolerance in vitro by autologous murine testicular cells. J Exp Med 151:827–838, 1980.
14. Richards JM. Bedford JM, Witkin SS: Rectal insemination modifies immune responses in rabbits. Science 224:390–392, 1983.
15. Witkin SS: Failure of sperm-induced immunosuppression: association with antisperm antibodies in women. Am J Obstet Gynecol 160:116–1168, 1989.
16. Munoz G, Posnett DN, Witkin SS: Enrichment of γδ T lymphocytes in human semen: relation between γδ T cell concentration and antisperm antibody status. J Reprod Immunol 22:47–57, 1992.
17. Munoz G, Witkin SS: Autoimmunity to spermatozoa, asymptomatic Chlamydia trachomatis genital tract infection and γδ T lymphocytes in seminal fluid from the male partners of couples with unexplained infertility. Human Reprod 10:1070–1074, 1995.
18. Mukasa A, Hiromatsu K, Matsuzaki G, O'Brien R, Born W, Nomoto K: Bacterial infection of the testis leading to autoaggressive immunity triggers apparently opposed responses of αβ and γδ T cells. J Immunol 155:2047–2056, 1995.
19. Fu YX, Roark CE, Kelly K, Drevets D, Campbell P, O'Brien R, Born W: Immune protection and control of inflammatory tissue necrosis by γδ T cells. J Immunol 153:3101–3115, 1994.
20. Suzuki T, Hiromatsu K, Ando Y, Okamoto T, Tomoda Y, Yoshikai Y: Regulatory role of γδ T cells in uterine intraepithelial lymphocytes in maternal antifetal immune response. J Immunol 154:4476–4484, 1995.
21. Hsieh B, Schrenzel MD, Mulvania T, Lepper HD, DiMolfetto-London L, Ferrick DA: In vivo cytokine production in murine listeriosis. Evidence for immunoregulation by $\gamma\delta^{+}$ T cells. J Immunol 156:232–237, 1996.
22. Hacker G, Adam D, Wagner H: Interaction between γδ T cells and B cells regulating IgG production. Immunol 84:105–110, 1995.
23. Hall TJ: Role of hsp70 in cytokine production. Experientia 50:1048–1053, 1994.
24. Jurivich DA, Pangas S, Qiu L, Welk JF: Phospholipase A2 triggers the first phase of the thermal stress response and exhibits cell- type specificity. J Immunol 157:1669–1677, 1996.
25. Schmidt JA, Abdulla E: Down-regulation of IL-1β biosynthesis by inducers of the heat-shock response. J Immunol 141:2027–2034, 1988.
26. Snyder YM, Guthrie L, Evans GF, Zuckerman SH: Transcriptional inhibition of endotoxin-induced monokine synthesis following heat shock in murine peritoneal macrophages. J Leukocyte Biol 51:181–187, 1992.
27. Munoz MG, Jeremias J, Witkin SS: The 60kD heat shock protein in human semen: relation to antibodies to spermatozoa and Chlamydia trachomatis. Human Reprod, (in press).
28. Nagasawa H, Hisaeda H, Maekawa Y, Fujioka H, Ito Y, Aikawa M, Himeno K: γδ T cells play a crucial role in the expression of 65,000 MW heat shock protein in mice immunized with Toxoplasma antigen. Immunol 83:347–352, 1994.
29. O'Brien RL, Fu YX, Crabfill R et al.: Heat shock protein Hsp60- reactive γδ T cells: A large, diversified T-lymphocyte subset with highly focused specificity. Proc Natl Acad Sci USA 89:4348–4352, 1992.
